# Temporal dynamics of sugar beet (*Beta vulgaris* L.) N supply from cover crops differing in biomass quantity and composition

**DOI:** 10.3389/fpls.2022.920531

**Published:** 2022-08-04

**Authors:** Heinz-Josef Koch, Dennis Grunwald, Lisa Essich, Reiner Ruser

**Affiliations:** ^1^Department of Agronomy, Institute of Sugar Beet Research, Göttingen, Germany; ^2^Department of Fertilization and Soil Matter Dynamics (340i), Institute of Crop Science, University of Hohenheim, Stuttgart, Germany

**Keywords:** nitrogen, winter rye, oil radish, spring vetch, bare fallow, net N mineralization, N effect, saia oat

## Abstract

Cover crops are supposed to decrease the soil mineral N content (N_min_) during winter and increase the N supply to subsequent main crops due to mineralization of N previously prevented from leaching. However, data on N supply from cover crops grown before sugar beet have rarely been reported for Central European conditions. Therefore, our study aimed to provide information for cover crops differing in frost resistance and biomass quantity applicable for N fertilizer dressing in the subsequent main crop. In 2018/19 and 2019/20, field trials were conducted on two Luvisol sites in Germany typical for sugar beet cultivation, comprising a sequence of autumn sown cover crops grown after field pea followed by unfertilized sugar beet main crops sown in next spring. Apparent net N mineralization and the N effect of cover crops on sugar beet were calculated according to a mass balance approach including N_min_ and sugar beet N uptake. Winter rye and oil radish revealed the greatest potential for scavenging nitrate from the soil profile while reductions caused by frost-sensitive saia oat and spring vetch were more variable. The amount of N in the cover crop biomass was negatively correlated with N_min_ in autumn and also in spring. Thus, for environmentally effective cover cropping in Central Europe, species with a sufficiently high frost tolerance should be chosen. Despite cover crop N uptake up to 170 kg N ha^−1^ and C:N ratios < 20, a positive N effect on sugar beet was only found between March and July of the beet growing season and was 50 kg N ha^−1^ at maximum, while between August and September, net immobilization was predominant with up to 100 kg N ha^−1^. Differences among crop species were not consistent across the site/years investigated. Sugar yield was lowest after rye at 3 sites/years and correlated positively with N_min_ in spring. Correlation between yield and cover crop N effect was mostly low and inconsistent and could not be improved by a multiple regression approach. Thus, factors other than in-season N supply from cover crops apparently impacted sugar beet yield formation to a larger extent.

## Highlights

- Frost-tolerant crop species produced highest biomass under various conditions.
- High cover crop biomass ensured low soil N_min_ in autumn and spring.
- N supply from cover crop N was positive in the first months of the sugar beet growing season, but negative afterwards.
- Whole season N effect to sugar beet was inconsistent among cover crops.
- Differences in soil N_min_ in spring caused by cover crops correlated satisfactorily with sugar yield.

## Introduction

Cover crops, which are also addressed as catch crops, are vital components of rotational cropping systems in regions where just one main crop can be grown per year due to winter coldness (Abdalla et al., 2019). In the temperate climate zone of Europe, cover crops are usually sown in August–September after previous main crop harvest, and growth is terminated either by frost killing in winter or by tillage or herbicide application next spring. Plant species grown as cover crops are mostly grasses, brassicas, and legumes which are seeded in pure stands or in species or variety mixtures. Farmers widely adopted cover crop cultivation in many parts of Europe, resulting in cover cropping previous to sugar beet on, e.g., 70% of fields in Germany in 2019 and >80% of fields in Northern France in 2012 (EUBSSP, 2016; Roß and Stockfisch, 2022). Nitrate retention (Thapa et al., 2018), carbon sequestration (Poeplau and Don, 2015), reduction of N_2_O release (Abdalla et al., 2019), increased arthropod biodiversity in soil (Fiorini et al., 2022) and erosion control (Blanco-Canqui et al., 2015) have been reported as environmental benefits of cover cropping. In addition, soil structural ameliorations (Haruna et al., 2020), enhanced rootability, and weed (Vincent-Caboud et al., 2019) and cyst nematode control (Hauer et al., 2016) are important targets of cover crop cultivation. Thereby, cover crops are expected to contribute to the goals of the EU Farm-to-Fork strategy, being part of the European Green Deal for a climate neutral continent (European Commission, 2021).

Moreover, the potential of cover crops to supply N to the subsequent main crop is of major importance for the adoption of cover cropping by farmers due to possible reductions in N fertilization. N transformation processes in soils grown with cover crops during their growing season, over the following winter, and during the growing period of the first and second subsequent main crops have been studied for a broad variety of conditions, as reviewed by, among others, Thorup-Kristensen et al. (2003), Dabney et al. (2010), and Sieling (2019). Cover crop effects on main crop N supply are quantified by changes in the total plant N content of the succeeding main crop. In addition to (net) mineralization and immobilization, differences in soil mineral N (N_min_) available for the main crop after winter (“pre-emptive competition”) might cause changes in the main crop N content. The overall difference between a specific cover crop treatment and bare fallow soil can be caused by both, changes in N_min_ and net N mineralization/immobilization during the main crop growing season; it was termed “N effect” by Thorup-Kristensen and Nielsen (1998).

Differences in the N effect (N_eff_) among cover crops depend on cover and main crop characteristics as well as on environmental conditions. For example, in his review of studies conducted on field sites in temperate climate, Sieling (2019) compiled N_eff_ for subsequent spring cereal, maize, and potato main crops in a range of −130 to +105 kg N ha^−1^; values were highest after leguminous cover crops and with low or zero N fertilization to the main crop, and lowest after frost-resistant grasses and high main crop N doses. Based on data from Thorup-Kristensen (1994), Thorup-Kristensen et al. (2003) reported N_eff_ values ranging from −22 up to 46 kg N ha^−1^ for spring barley grown after a broad variety of cover crops (brassicas, grasses, legumes; frost-resistant or not). The lowest value was obtained from rye grass and the highest from radish cover crop. Even more variable values were found for N_eff_ of winter rye cover crop preceding spring barley in the study of Thorup-Kristensen and Dresbøll (2010) with values of −100 up to 70 kg N ha^−1^. Very low values occurred in this study after a dry winter, when high amounts of soil N_min_ were retained in the absence of a cover crop due to low leaching losses. Consequently, a high amount of N_min_ was readily available for the barley crop after fallow while after rye cover crop N captured by rye biomass had to be mineralized before becoming available. Thus, the amount of water percolating over winter and moreover, the amounts of N and C assimilated by cover crops are strongly affecting N_eff_ as governing factors of a later mineralization (Thorup-Kristensen and Dresbøll, 2010).

Compared to cereals, sugar beet growth in spring is slow, and the initial crop N uptake accounts to only a few kg N ha^−1^ until the end of May, increasing sharply to 80 kg N ha^−1^ until mid-June and >200 kg N ha^−1^ in August for Central European conditions (Windt, 1995). Later in the season, changes in total plant N content are often small. At autumn harvest, N content of beet crops grown under optimum N supply conditions was found to be in the range of 200–250 kg N ha^−1^, which was widely independent of the sugar yield level (Hoffmann et al., 2020). Despite its low initial N demand in absolute terms, adequately high N supply is crucial for rapid canopy development, maximum light interception and, consequently, a high yield (Hoffmann and Kluge-Severin, 2010). Sugar beet crops can satisfy their N need from soil N_min_ in early spring and N mineralization, which amounts to up to 160 kg N ha^−1^ on silty loam soil, with mineralization rates increasing from sowing in April to June followed by lower values later in the season (Hoffmann et al., 1996). Cover crops are supposed to increase N supply to the subsequent beet crop compared to fallow due to mineralization of N previously prevented from leaching. However, data on N supply from cover crops grown before sugar beet have rarely been reported for Central European conditions. In this regard, cover crop types differing in biomass, dry matter composition, and frost resistance, all affecting their N uptake and thus N_min_ in spring, are expected to result in different amounts of N supply to sugar beet; in consequence, cover crop choice might be relevant for the crop's yield formation.

Our study aimed to provide information on the seasonal N supply from different cover crop species types on sites typical for sugar beet cropping in Central Europe. Thereby, basic information for an adequate N fertilizer dressing after cover crops should be gained. The investigated cover crops were the grass species saia oat (*Avena strigosa* Schreb.) and winter rye (*Secale cereale* L.), and the herbaceous plants oil radish (*Raphanus sativus* var. *oleiformis* Pers.) and finally spring vetch (*Vicia sativa* L.) as legume. The two grasses and radish were chosen because of their assumedly wide C:N ratio, and high capability for dry matter formation and thus removal of N_min_ in autumn. Frost-resistant rye was expected to maintain soil N_min_ at a low level until spring, while the N taken up by frost-sensitive oat and vetch should be re-mineralized at an earlier stage.

We hypothesized that, compared to bare fallow, cover crops (i) decrease soil N_min_ in early spring, (ii) increase the N supply to subsequent unfertilized sugar beet main crop (N_eff_), and that (iii) the net effect of these two processes on sugar beet N uptake and sugar yield is close to zero as they are contrary to each other. We further hypothesize that (iv) the decrease of spring N_min_ and the increase of N supply to sugar beet after cover crops compared to fallow is higher for cover crops with a large C and N contents. Such effects may be modified by the C:N ratio in the cover crop biomass and overwinter N leaching.

Field trials were conducted at two locations in two subsequent years starting 1 year offset. The locations were chosen as representative for sugar beet cultivation in large parts of Central Europe.

## Materials and methods

### Trial sites and experimental design

The trial sites were located at Ihinger Hof (Renningen, Southwest Germany; 48.7469714, 8.92351448; 480 m asl; Iho18/19 and Iho19/20) and near Göttingen (Central Germany; 51.6370104, 9.8931940; 160 m asl; Goe18/19 and Goe19/20). Different fields were used at both locations for subsequent years so that no residual effects of the trials 2018/19 are present in the trials 2019/20. At both sites, the soil was classified as Luvisol. Topsoil (0–30 cm) clay contents were 255–285 and 119–143 g kg^−1^ and silt contents 697–714 and 709–849 g kg^−1^, at Ihinger Hof and Göttingen, respectively. At both sites, the topsoil organic matter content was 20–30 g kg^−1^, and the pH (0.0125 m CaCl_2_) was pH 7.0–7.5; the gravel content was zero. Long-term annual precipitation and mean air temperature are 738 mm a^−1^ and 8.4°C at Iho and 624 mm a^−1^ and 9.5°C at Goe (DWD, 2022). In the main cover crop growing period (August–November) of the experimental years, the air temperature was 0.6–1.6°C higher than the long-term average (Iho 10.7°C, Goe 11.4°C) and annual precipitation was 22–105 mm lower than average (Iho 230 mm, Goe 206 mm). Differences were more pronounced in 2018 than in 2019. In Table 1, rainfall and temperature characteristics are displayed for three periods: (i) over winter, beginning after the cover crop season until N_min_-sampling in March, (ii) from March to an in-season sugar beet sampling date, which was in July or August, depending on site and year, and (iii) from the sampling date in July/August to sugar beet harvest which took place between the end of September and the end of October. Differences in sampling and harvest dates between the sites were caused by largely different weather conditions. Thus, the data presented differ in period length and position in the season.

**Table 1:** Cumulated rainfall and mean and sum of air temperature for three sub-periods from cover crop sampling until sugar beet harvest at Ihinger Hof and Göttingen in 2018/19 (Iho18/19, Goe18/19) and 2019/20 (Iho19/20, Goe19/20).

**Site**	**November–March**				**March–July/August**				**July/August–September/October**			
	**Rain**	**Air temp.**		**Sampl.**	**Rain**	**Air temp.**		**Sampl.**	**Rain**	**Air temp.**		**Sampl.**
	**(mm)**	**Mean (°C)**	**Sum (°C d)**	**Date**	**(mm)**	**Mean (°C)**	**Sum (°C d)**	**Date**	**(mm)**	**Mean (°C)**	**Sum (°C d)**	**date**
Iho18/19	106	2.6	225	3/29/19	322	14.5	2,219	08/28/19	79	13.4	764	10/24/19
Iho19/20	142	4.0	336	3/25/20	158	12.9	1,567	07/23/20	114	17.9	1,200	09/29/20
Goe18/19	317	4.0	518	3/12/19	217	13.0	1,585	07/12/19	89	18.1	1,342	09/25/19
Goe19/20	472	4.8	643	3/16/20	255	12.8	1,568	07/16/20	174	17.8	1,372	10/01/20

Weather record started on 1st of November and the first period was terminated at the final soil sampling date before sowing. All subsequent periods ended with a sampling of sugar beet plants and soil N_min_.

Rain, rainfall; Air temp., air temperature; Sampl., sampling.

At both sites, combine-harvested field pea (*Pisum sativum* L.) was grown as preceding crop. Pea straw was left in the field and incorporated into the soil by plowing or deep rigid tine cultivator tillage shortly before cover crop sowing. The experiments had a two-factorial randomized split-plot design with four replicates organized in complete blocks. Five cover crop treatments served as main plots (30.0 × 19.0 m at Ihinger Hof, 21.0 × 17.0 m at Göttingen), consisting of (i) bare fallow control, (ii) oil radish, (iii) saia oat, (iv) spring vetch, and (v) winter rye, while four different N fertilization levels of sugar beet served as subplots (3.0 × 12.0 m at Ihinger Hof and 2.7 × 14.0 m at Göttingen). For this study, only subsequent sugar beet plots were considered which received no N fertilization.

Cover crops were sown on September 12, 2018 and September 4, 2019 at Iho, and on August 29, 2018 and August 8, 2019 at Goe after seedbed preparation with a disc or rotary harrow. The cover crop varieties were Defender, Pratex, Mirabella, and Traktor (Federal Plant Variety Office, 2018) for radish, oat, vetch, and rye, respectively, sown with a seed rate of 30, 80, 90, and 120 kg ha^−1^, respectively, and a row spacing of 15.0 cm at Iho and 12.5 cm at Goe. The bare fallow treatment was kept free of volunteers and weeds by one or two herbicide sprayings in autumn. The cover crops developed well to dense stands and successfully suppressed upcoming weeds and volunteers. In January 2019, radish, oat and vetch plants were killed by frost at both sites. At Iho19/20, cover crops were not affected by frost over winter; at Goe19/20, frost at the end of November 2019 severely damaged vetch, while oat died because of senescence and radish survived without frost damage. Rye survived winter at both sites and years, and all cover crop plots were sprayed with glyphosate in March. Afterwards, soil tillage was performed by moldboard plowing to 30 cm depth in the rye plots and short disc harrowing or rigid tine cultivator tillage to 15 cm depth in all other plots.

Seedbed preparation for cover crops and sugar beet was performed with a rotary harrow at Iho and a light tine harrow in Goe. Sugar beet was sown in six row plots on April 16 in both 2019 and 2020 at Iho, and April 9, 2019 and April 3, 2020 at Goe. Row spacing was 50 cm at Iho and 45 cm at Goe, and plant density was 90.000–95.000 plants ha^−1^.

### Plant and soil analyses and calculations

Aboveground biomass of cover crops was determined by the end of November at each site/year combination on four points of 0.5 m^2^ per main plot. Fresh biomass of sugar beet taproots and leaves was determined in July/August (refer Table 1 for sampling dates) on 4.5 and 5.4 m^2^ per plot at Iho and Goe, respectively, while for the September/October harvest, plot size was 10.4–16.0 m^2^, depending on site and year. Dry matter content of the cover crop biomass and the two sugar beet fractions was determined by drying a mashed subsample at 60°C for 48 h and used to calculate dry matter biomass. Plant material was analyzed for C and N concentrations by elementary analysis; concentrations were used for the calculation of the C and N contents of aboveground cover crop biomass and total beet plants. The C content of the cover crops is presented instead of the cover crop biomass since C, in combination with N, is the element which is mainly affected by biomass decomposition in quantitative and qualitative terms and moreover, strongly impacts N release. In addition, C content and dry matter biomass strongly correlated for the cover crops with *r* > 0.9 except at Iho18/19 where the correlation was less close.

Sugar concentration of the taproot brei was analyzed polarimetrically (ICUMSA, 1994), and the sugar yield was calculated by multiplication with the clean taproot yield.

Soil samples from 0.0–0.3, 0.3–0.6, and 0.6–0.9 m soil depth were taken as composite samples of 3–5 subsamples from each plot in monthly intervals from November to April, and at the plant sampling dates in July/August and September/October to determine soil mineral N (N_min_, nitrate + ammonium); 20–50 g of fresh soil were extracted with 0.0125 *M* CaCl_2_ solution (ratio 1:4) (VDLUFA, 2020). In July 2019, sampling below 0.3 m depth was impossible due to extremely dry and rigid soil conditions at Göttingen. Therefore, N_min_ was assumed as 20 kg N ha^−1^ because data from the other site/year combinations showed that sugar beet had almost completely depleted the profile of the deep-rooted Luvisol soils until mid–July.

For all treatments, net N mineralization (NetN-Min) was calculated for the periods from (i) N_min_ soil sampling in March to sampling in July/August and (ii) sampling in July/August to harvest in September/October, and was furthermore summed up for the complete growing season. Sampling in March was chosen as starting point because this date is the regular sampling date for determining the fertilizer N demand of sugar beet crops in Europe and sufficiently close to the sowing date. NetN-min in a distinct period was calculated according to a mass balance approach (Köhler, 1983):


(1) $$\begin{array}{l} NetN-Min=(N\;content_{t2}\;-N\;content_{t1})\\ \;+(N_{min}\_0.0-0.9m_{t2}-N_{min}\_0.0-0.9m_{t1}) \end{array}$$

where N content is the N content of the complete sugar beet plant at a distinct date t and N_min__0.0–0.9m is the N_min_ content of the soil profile down to 0.9 m at the same distinct date, with all values in kg N ha^−1^. The indices t1 and t2 indicate the date of sampling. In this approach, it is assumed that dentrification equals N deposition and N_2_ fixation, and that N leaching does not occur (Köhler, 1983). The NetN-Min for the period July/August to harvest was calculated as the difference of the values for the periods March to harvest and March to July/August.

According to the definition given by Thorup-Kristensen and Nielsen (1998), the N effect (N_eff_, kg N ha^−1^) of a cover crop for subsequent sugar beet for the periods March–July/August and March–September/October was calculated as the difference between the total plant N content of sugar beet grown after a specific cover crop (CC) and the bare fallow (BF) treatment (kg N ha^−1^):


(2) $$\begin{array}{r} {\text{N}}_{\text{eff}}\;=\text{ N content CC }-\text{ N content BF} \end{array}$$

N_eff_ for the period from July/August until September/October was calculated by deducting N_eff_ for the period March–July/August from N_eff_ for the period March–September/October.

### Statistical analyses

The statistical data evaluation was carried out with SAS Version 9.4 (SAS Institute Inc., Cary, NC, United States). Analyses were performed separately for each site/year combination due to considerable differences in treatment effects among site/year combinations. Cover crop treatment effects on aboveground C and N contents and sugar yield were analyzed by a mixed model (proc mixed) ANOVA with cover crop treatment as fixed effect and block as random effect. Two-factorial ANOVA were performed for the dependent variables sugar beet N content, NetN-Min and N_eff_ with sampling date (sugar beet N content) or sampling period (NetN-Min, N_eff_) as first factor and cover crop treatment as second factor, as well as their interaction. For the factor sampling date/period, the “*repeated*” statement was used, and replicates were assigned as nested within sampling date/period.

Cover crop effects on N_min_ content were analyzed separately for each sampling date. In a second step, differences in N_min_ content between sampling dates/periods were tested for significance separately for each cover crop, by applying the “*repeated*” statement and nesting replicates within sampling date/period. Thereby, clarity and comprehensibility of data presentation were sustained although in some cases *p*-values for the differences between means slightly differed from the two-factorial analysis.

Residuals of the models were checked for homoscedasticity by Levene's test and graphically, and for normal distribution by Shapiro-Wilk's test as well as graphically. If the respective factor (cover crop treatment, sampling date/period) revealed to be significant (*p* < 0.05), least square means were compared by calculating least significant differences by Tukey-Kramer's test.

To evaluate the correlations among cover crop biomass properties and N_min_ in November and March, NetN-Min, and N_eff_, Pearson's correlation coefficients were calculated by SAS proc corr. In this analysis, fallow treatment was left out because it provided no cover crop biomass data. Further, correlations between sugar yield and spring N_min_ and NetN-Min including the fallow treatment were calculated. In a multiple linear regression analysis using sugar yield as dependent variable, N_min_ in March, NetN-Min, and N_eff_ were used as explanatory variables separately for data including (N_min_, NetN-Min) and excluding (N_min_, N_eff_) bare fallow treatment. Initially, all parameters revealing a significant correlation with the sugar yield were included followed by a stepwise removal of insignificant parameters from the model in a backward approach.

## Results

The C and N contents of cover crops ranged between 365–1,658 kg C ha^−1^ and 41–172 kg N ha^−1^, respectively (Table 2). At all site/year combinations, values were high for radish and rye, while vetch and oat had both high and low values at different site/year combinations. The C:N ratio of cover crop biomass was lowest for vetch in all cases (<10), clearly wider with up to 17 for oat and rye, and intermediate for radish (Table 2).

**Table 2:** C and N content and C:N ratio of aboveground biomass of four cover crops in late autumn at Iho18/19 and 19/20, and Goe18/19 and 19/20.

**Cover crop**	**Iho18/19**	**Iho19/20**	**Goe18/19**	**Goe19/20**
**C content (kg C ha** ^**−1**^ **)**				
Oil radish	904 a	1,324 a	979 b	1,181 a
Saia oat	949 a	958 b	1,140 a	687 c
Spring vetch	365 b	656 b	667 c	880 bc
Winter rye	1,096 a	1,658 a	1,187 a	1,032 ab
**N content (kg N ha** ^**−1**^ **)**				
Oil radish	74	141 a	104 a	111 a
Saia oat	61	82 b	104 a	50 c
Spring vetch	41	78 b	77 b	92 b
Winter rye	65	172 a	109 a	99 ab
**C:N ratio (-)**				
Oil radish	12.8*bc*	9.4*b*	9.4*b*	10.6*b*
Saia oat	15.7*ab*	11.7*a*	10.9*a*	13.6*a*
Spring vetch	9.0*c*	8.4*c*	8.6*b*	9.6*b*
Winter rye	17.1*a*	9.6*b*	11.0*a*	10.4*b*

Means of 4 replicates.

Different letters indicate significant differences among cover crops within individual site/year combinations at p < 0.05 (Tukey-Kramer).

**Figure 1:**
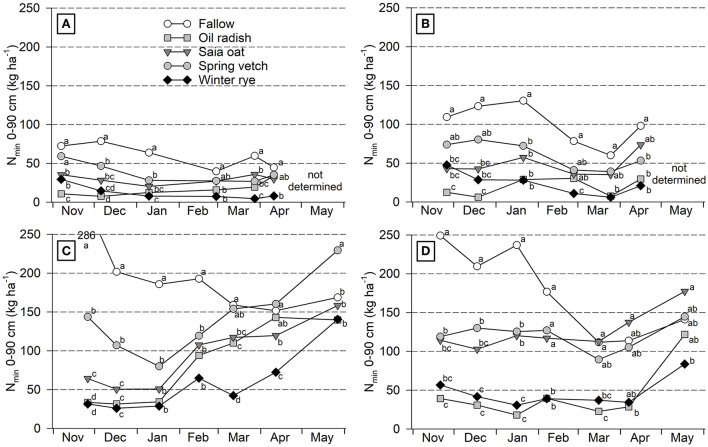
Course of soil N_min_ over winter and during early growth of unfertilized sugar beet as affected by different cover crop treatments at Ihinger Hof 2018/19 **(A)** and 2019/20 **(B)**, and Göttingen 2018/19 **(C)** and 2019/20 **(D)**. Mean of 4 replicates. At each site/year combination different letters within one sampling date indicate significant differences at *p* < 0.05 (Tukey-Kramer).

Soil N_min_ values were overall low at Iho18/19, substantially higher at Goe18/19 and 19/20, and intermediate at Iho19/20 (Figure 1; Supplementary Figure S1). In most cover crop treatments and site/year combinations, N_min_ decreased from November to sampling in January–March. The decrease was biggest if the initial N_min_ was high such as for the fallow treatment at Goe18/19 and 19/20; however, values often remained constant during winter, if N_min_ was low in November already. Lowest values occurred under radish and rye while under vetch and oat N_min_ was intermediate, except for oat at Goe18/19 where it was similarly low as for radish and rye. After winter, N_min_ significantly increased in some treatments at three of the four site/year combinations, starting in January at Goe18/19 and in March at Goe19/20 and Iho19/20. Between the end of March and May lowest values were found after rye which were in some cases significantly lower than after fallow, vetch, or oat.

At plant sampling in July/August, sugar beet N content ranged from 111 to 182 kg N ha^−1^ and was lowest after fallow and rye at both sites in 2018/19, while in 2019/20 differences among cover crop treatments were less pronounced (Table 3). At the September/October harvest, N content had significantly increased compared to July/August to ≥250 kg N ha^−1^ at Goe19/20, whereas for the other site/year combinations, the increase was much smaller in most treatments and N content was ≤250 kg N ha^−1^. Remarkably, in some site/year/cover crop combinations, the increase in N content in the period July/August–September/October was <15 kg N ha^−1^ (e.g., Iho18/19, radish and oat) while in other cases, it was >150 kg N ha^−1^ (Goe19/20, vetch). In general, sugar beet N content at harvest was strongly correlated with sugar beet biomass (data not shown; *r* = 0.85).

**Table 3:** N content of unfertilized sugar beet (kg N ha^−1^) after different cover crop treatments at two dates of the growing season at Ihinger Hof 2018/19 and 2019/20, and Göttingen 2018/19 and 2019/20.

	**July/August**	**September/October**	**July/August**	**September/October**
	**Ihinger Hof 2018/19**		**Ihinger Hof 2019/20**	
Fallow	117 c	199 a	135 c	251 a
Oil radish	153 abc	164 abc	160 abc	164 abc
Saia oat	163 abc	166 abc	126 c	231 ab
Spring vetch	152 abc	192 ab	156 abc	249 a
Winter rye	126 c	143 bc	119 bc	168 abc
	**Göttingen 2018/19**		**Göttingen 2019/20**	
Fallow	129 ef	259 a	148 d	297 ab
Oil radish	133 ef	178 bcd	150 d	239 c
Saia oat	141 def	203 bc	158 d	284 abc
Spring vetch	182 bcd	224 ab	154 d	324 a
Winter rye	111 f	165 cde	161 d	262 abc

Means of 4 replicates.

Different letters indicate significant differences among cover crops across both sampling dates within single site/year combinations at p < 0.05 (Tukey-Kramer).

**Figure 2:**
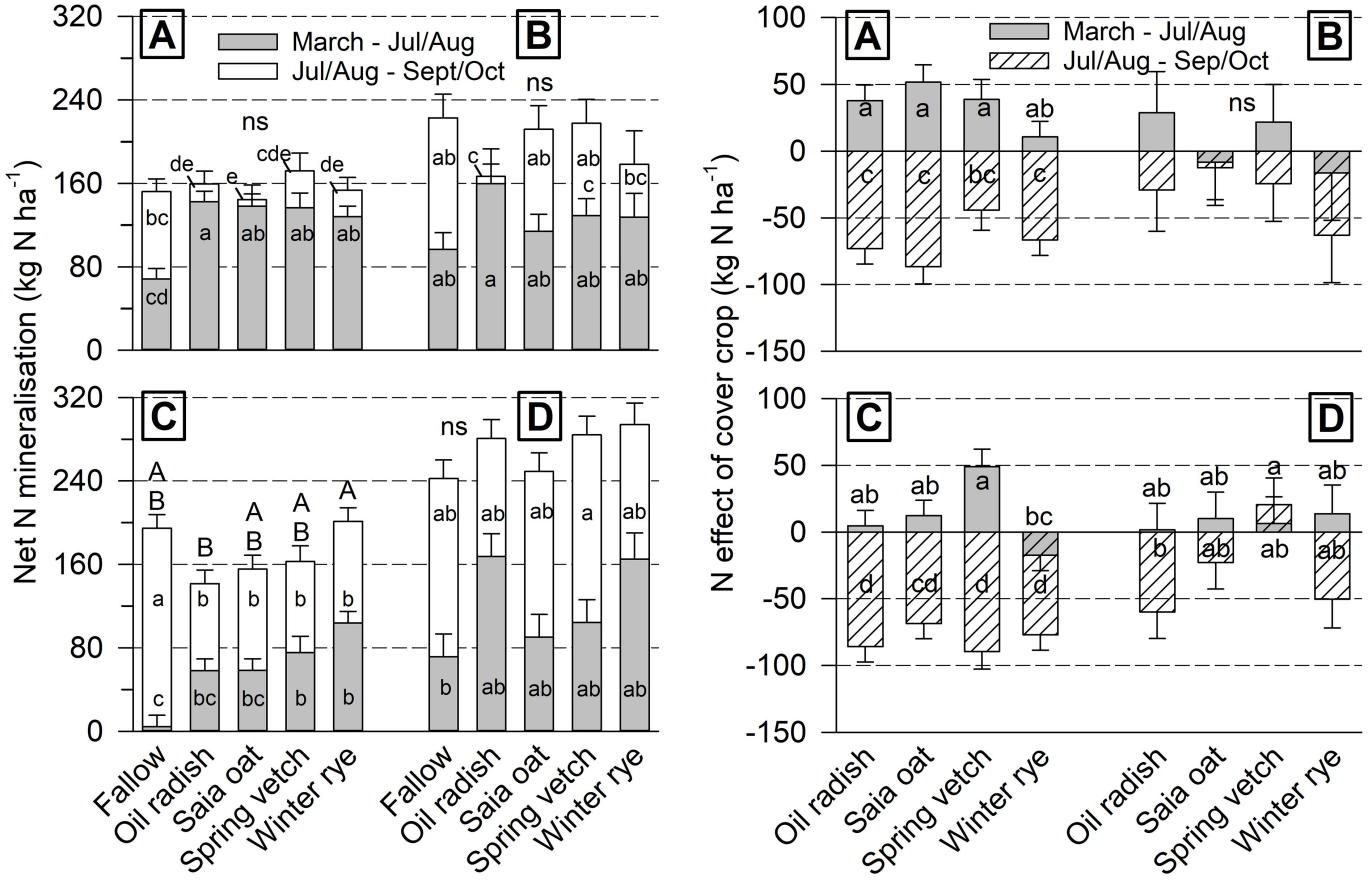
Cover crop treatment effect on soil net N mineralization (left) and cover crop N effect (right) under unfertilized sugar beet in two periods of the growing season (mean of 4 replicates ± standard error) at Ihinger Hof 2018/19 **(A)** and 2019/20 **(B)**, and Göttingen 2018/19 **(C)** and 2019/20 **(D)**. Different lowercase letters indicate significant differences among cover crop treatments within single site/year combinations at *p* < 0.05 (Tukey-Kramer). Uppercase letters refer to the whole season from March to final harvest, ns not significant.

Whole season NetN-Min amounted to about 160 kg N ha^−1^ at both sites in 2018/19 (Figure 2). At Iho19/20, it ranged between 160 and 240 kg N ha^−1^, and at Goe19/20 between 240 and 300 kg N ha^−1^. Whole season values differed among cover crops only at Goe18/19, where NetN-Min was significantly higher after rye compared to radish, whereas for the other cover crop treatments it was intermediate. In 2019, NetN-Min after fallow was lowest for the period March–July/August, but highest for the period July/August–September/October. In 2020, this effect was not found.

In the period March–July/August, the cover crop N_eff_ was positive with values up to 50 kg N ha^−1^ or just slightly negative (−15 kg N ha^−1^) at all site/year combinations (Figure 2). Significant differences in N_eff_ among cover crops occurred in 2018/19 at both sites with lowest values after rye and radish (Goe18/19 only). In the period July/August–September/October, N_eff_ was negative in a range of −10 to −100 kg N ha^−1^, with the exception of vetch at Goe19/20. However, differences among cover crops were not significant at any site/year combination for this period.

**Figure 3:**
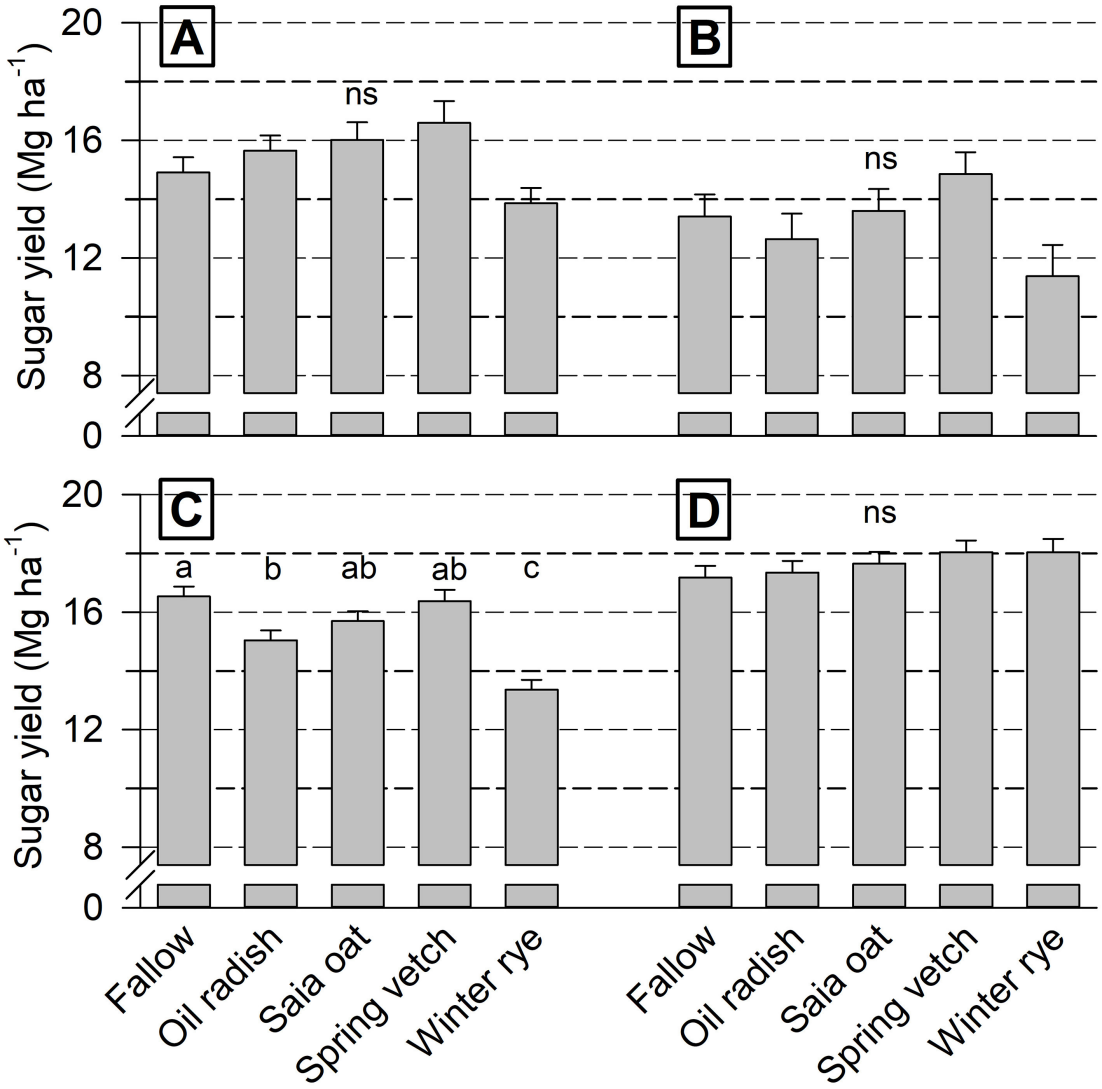
Cover crop treatment effect on sugar yield of unfertilized sugar beet (mean of 4 replicates + standard error) at Ihinger Hof 2018/19 **(A)** and 2019/20 **(B)**, and Göttingen 2018/19 **(C)** and 2019/20 **(D)**. Different letters indicate significant differences among cover crop treatments within each site/year combination at *p* < 0.05 (Tukey-Kramer), ns not significant.

Overall, sugar yield level was highest at Goe19/20 and lowest at Iho19/20 (Figure 3). Differences among cover crops were significant at Goe18/19 only, with yield being highest after fallow and vetch and lowest after rye. A tendency toward a lower yield after rye and a higher yield following vetch was also found at Iho18/19 and 19/20.

At all site/year combinations, autumn and spring N_min_ were significantly negatively correlated with cover crop C and N contents, except for spring N_min_ at Iho18/19 (Table 4). NetN-Min of the periods July/August–September/October and March–September/October was negatively correlated with cover crop C and N content at Iho19/20. In contrast, correlations were positive for March–July/August at Goe19/20. In 2018/19, no correlation between cover crop characteristics and net mineralization was found at either site. Cover crop N effect was negatively correlated with C and N content for most periods and site/year combinations; however, only few coefficients were significant. At Iho19/20 and Goe18/19, the correlation between sugar yield and cover crop C and N content, respectively, was negative, while at the other site/year combinations these parameters were not correlated with sugar yield.

**Table 4:** Pearson correlation coefficients for the correlation among cover crop C and N content and soil N_min_, net N mineralization under following unfertilized sugar beet (NetN-Min) and the cover crop N effect on sugar beet (N_eff_) for different dates/periods, and autumn sugar yield at Ihinger Hof 2018/19 and 2019/20, and Göttingen 2018/19 and 2019/20.

	**C content (kg ha^−1^)**	**N content (kg ha^−1^)**	**C content (kg ha^−1^)**	**N content (kg ha^−1^)**
	**Ihinger Hof 18/19**		**Ihinger Hof 19/20**	
N content	**0.74**		**0.99**	
N_min_Nov_0-90 cm	*−0.55*	*−0.55*	−**0.56**	*−0.48*
N_min_Mar_0-90 cm	−0.33	−0.08	−**0.56**	−**0.60**
NetN-Min_Mar-Jul/Aug	0.01	0.24	0.08	0.20
NetN-Min_Jul/Aug-Sep/Oct	−0.09	0.13	*−0.54*	−**0.61**
NetN-Min_Mar-Sep/Oct	−0.10	0.34	*−0.45*	−0.43
Neff_Mar-Jul/Aug	−0.42	−0.19	−0.16	0.00
Neff_Jul/Aug-Sep/Oct	*−0.55*	−0.36	−0.30	−0.33
Neff_Mar-Sep/Oct	−**0.71**	−0.42	−0.30	−0.26
Sugar yield	0.40	0.07	−**0.59**	*−0.51*
	**Göttingen 18/19**		**Göttingen 19/20**	
N content	**0.85**		**0.93**	
N_min_Nov_0–90 cm	−**0.81**	−**0.83**	−**0.69**	−**0.62**
N_min_Mar_0–90 cm	−**0.75**	−**0.64**	−**0.74**	−**0.72**
NetN-Min_Mar-Jul/Aug	0.31	0.27	**0.61**	**0.62**
NetN-Min_Jul/Aug-Sep/Oct	−0.08	−0.31	−0.26	−0.27
NetN-Min_Mar-Sep/Oct	0.22	0.04	0.33	0.33
Neff_Mar-Jul/Aug	−**0.74**	−**0.63**	−0.15	−0.11
Neff_Jul/Aug-Sep/Oct	0.28	0.13	−0.26	−0.18
Neff_Mar-Sep/Oct	*−0.48*	*−0.49*	−0.44	−0.31
Sugar yield	−**0.65**	−**0.53**	−0.01	−0.03

Coefficients≥ |0.56|/|0.47| and |0.51|/|0.44| differ significantly from zero at p < 0.05/p < 0.10 at Ihinger Hof (N = 13) and Göttingen (N = 15), respectively. They are typed bold/italics.

Sugar yield correlated positively with spring N_min_ at Iho18/19, Iho19/20 and Goe18/19 (Table 5). Further, the correlation between N_eff_ and sugar yield was significantly positive for March–September/October at Goe18/19 and Goe19/20, and March–July/August at Iho18/19 and Goe18/19 (Table 5). For the correlation between sugar yield and NetN-Min, coefficients were significantly positive in the period July/August–September/October at Iho19/20, Goe18/19 and Goe19/20, and in March–September/October at both, Iho19/20 and Goe19/20. At Goe18/19, however, sugar yield was negatively correlated with NetN-Min in March–July/August. The combination of explanatory variables in the multiple regression analysis revealed no significant increase in *R*^2^ compared to single parameters with the highest *R*^2^ (not shown).

**Table 5:** Pearson correlation coefficients for the correlation among sugar yield and soil mineral N content (N_min_) in March, net N-mineralization (NetN-Min) under following unfertilized sugar beet, and the cover crop N effect on sugar beet (N_eff_) for different periods at Ihinger Hof (Iho) and Göttingen (Goe), both in 2018/19 and 2019/20.

	**Iho**	**Iho**	**Goe**	**Goe**
	**18/19**	**19/20**	**18/19**	**19/20**
**Excluding fallow treatment**				
N_min_March_0–90 cm	**0.82**	**0.66**	**0.85**	−0.03
Neff_Mar-Jul/Aug	*0.52*	0.43	**0.80**	0.06
Neff_Jul/Aug-Sep/Oct	−0.06	0.35	−0.15	0.41
Neff_Mar-Sep/Oct	0.21	0.46	**0.64**	**0.57**
**Including fallow treatment**				
N_min_Mar_0–90 cm	0.32	**0.48**	**0.88**	−0.17
NetN-Min_Mar-Jul/Aug	0.14	0.20	−**0.60**	−0.01
NetN-Min_Jul/Aug-Sep/Oct	0.10	**0.58**	**0.48**	**0.47**
NetN-Min_Mar-Sep/Oct	0.37	**0.79**	−0.04	**0.65**

For parameters including the bare fallow treatment, coefficients≥ |0.48|/|0.42| (N = 17) and |0.45|/|0.41| (N = 19) differ significantly from zero at p < 0.05/p < 0.10 at Ihinger Hof and Göttingen, respectively. For parameters using bare fallow as reference, coefficients ≥ |0.56|/|0.47| (N = 13) and |0.51|/|0.44| (N = 15) differ significantly from zero at p < 0.05/p < 0.10 at Ihinger Hof and Göttingen, respectively. They are typed bold and italics.

## Discussion

### Cover crop effects on N_min_ in autumn and over winter

In our field trials, field pea was grown as main crop preceding cover crops to cause high N mineralization and, consequently, high soil N_min_ in autumn. Without cover crops N_min_ in November was highest at Goe18/19 and 19/20 with values up to more than 250 kg N ha^−1^ in 0–0.9 m soil depth, which was likely due to favorable temperature conditions above long-term average, fostering N mineralization. At all site/year combinations, cover crops substantially reduced soil N_min_ by the end of November, which confirms previous studies (e.g., Abdalla et al., 2019). Oil radish and winter-hardy rye were most effective and constant in the reduction of soil N_min_ across site/year combinations, presumably due to their capability to (i) produce high biomass and take up high amounts of N mineralized in autumn, even under less favorable conditions (i.e., late sowing, drought, and cold in autumn) and (ii) resist frost events occurring in autumn. This confirms previous observations reported by, e.g., Thorup-Kristensen (1994), Thorup-Kristensen et al. (2003), Hashemi et al. (2013), and Sieling (2019).

By the end of November, the differences in soil N_min_ between fallow on the one hand and radish and rye on the other hand accounted for about 80 and 120 kg N ha^−1^ at Iho18/19 and 19/20, respectively, and 200 kg N ha^−1^ at Goe18/19 and 19/20. The N content of radish and rye was similar to the difference in N_min_ between these cover crops and fallow at Iho18/19 and tended to be 30–50 kg N ha^−1^ higher at Iho19/20. Contrastingly, at Goe18/19 and 19/20, the N content of radish and rye amounted to only 50% of the decrease in N_min_ by such crops. Diverse causes such as N stored in belowground biomass and N immobilization due to root exudates might be responsible for such discrepancies (Breland and Bakken, 1991; Böldt et al., 2021). Autumn leaching, as another potential cause, can be excluded in our experiments due to low rainfall amounts during the main cover crop growing season until November.

In the experiments 2018/19, N_min_ after cover crops was lowest in January 2019 at both sites. From January until March 2019, values remained constant or increased, slightly so at Iho18/19 and considerably at Goe18/19. This difference between the sites might be caused by a clearly higher temperature and thus higher mineralization in this period at Goe18/19 compared to Iho18/19. In contrast, after fallow, leaching due to high rainfall likely caused a continuous decrease in N_min_ from November/December until March which was more pronounced at Goe18/19 compared to Iho18/19 due to the threefold higher rainfall in this period. As a result of the increase in N_min_ after not frost-resistant cover crops due to mineralization and the decrease in N_min_ after fallow due to leaching, N_min_ values among the treatments converged until March, with differences up to 50–70 kg N ha^−1^ remaining. When considering the sum of overwinter rainfall, our data confirm that amounts <400 mm largely maintain differences in autumn N_min_ caused by cover cropping until spring, as previously stated by Thorup-Kristensen et al. (2003). However, the occurrence of leaching also in the cover crop treatments cannot be excluded in our study.

In 2020, minimum N_min_ values occurred in March, 2–3 months later than in 2019. At Iho19/20, N_min_ after fallow decreased from January to March as opposed to constant values in 2019, which might be due to the opposed effect of the substantially higher temperature and slightly higher rainfall in 2020 than 2019, thereby causing higher mineralization and some leaching. At Goe19/20, N_min_ values considerably decreased over winter after fallow treatment, which was likely because temperature sum and rainfall were highest at Goe19/20 among all site/year combinations; after cover crops, however, N_min_ remained constant, indicating that a higher N mineralization might have compensated for leaching.

Overall, our data show that (i) above-ground C and N content of cover crops in autumn correlated negatively with N_min_ in November and March, indicating that well-growing cover crop stands substantially reduce the risk for N leaching, and (ii) the order among cover crop treatments regarding N_min_ at the end of the growing season is maintained until March of the following year, even though on a lower level. However, this is only the case if rainfall is low in relation to water storage capacity of the soil and cover crops are sufficiently winter-hardy.

### Effect of cover crops on N mineralization and N supply to sugar beets

Mass balance methods to determine NetN-Min from organic amendments have been previously used by, e.g., Engels and Kuhlmann (1993) and Constantin et al. (2011). A comparison of this method with the estimation of N mineralized from undisturbed soil samples incubated *in situ* in polyethylene bags under sugar beet by Hoffmann et al. (1996) revealed a good agreement between the methods in one season and values twice as high for the soil incubation method in another season. Several causes can bias either method, such as atmospheric deposition, plant root exudation causing N immobilization, dentrification and, specifically for the balance method, severe leaf losses owing to leaf disease occurrence or drought stress, which would exclude N from detection either as plant N or N_min_. In our study, the growing seasons at Iho19/20 and Goe18/19 were characterized by low rainfall (<300 mm) and high temperatures compared to the other site/year combinations, hinting at possible drought stress for sugar beet. Nevertheless, the increase in sugar beet N content from mid-summer to autumn in the fallow treatment was reasonably high (>80 kg N ha^−1^) under all conditions. Thus, we can exclude drought causing strong bias to our data.

Our experiments were conducted on fertile Luvisol soil with a high potential for N mineralization during the growing season. We calculated amounts of N mineralized throughout the sugar beet growing season in a range of 160–280 kg ha^−1^ N with highest values under the favorable weather conditions of 2020. For similar conditions, up to 200 kg ha^−1^ N were also found by Engels and Kuhlmann (1993), Allison et al. (1996), Hoffmann et al. (1996), and Constantin et al. (2011).

Across all site/year combinations, the calculated NetN-Min in the period from March to July/August was lower after fallow compared to the treatments with cover crops. However, as N_min_ in March/April was mostly higher after fallow than after the cover crops (only in some cases significant), the N content of sugar beet in July/August was very similar in most treatments. As an exception, rye caused a somewhat lower sugar beet N content at sampling in both, July/August and September/October, at three out of four site/year combinations. Considering that NetN-Min was similar among cover crops, the decreased N uptake after rye was presumably due to the lower initial level of soil N_min_ in early spring, which was not compensated for by timely N release from rye biomass thereafter. Delayed or lower N mineralization resulting in lower N uptake of succeeding main crops after winter-hardy cover crops killed late in spring compared to early frost-killed cover crops or main crops grown without cover crops has also been reported by Wagger (1989), Thorup-Kristensen and Dresbøll (2010), and Hashemi et al. (2013), while Böldt et al. (2021) reported mixed results. As reviewed by, e.g., Thorup-Kristensen et al. (2003) and Sieling (2019), cover crop N supply to succeeding main crops is affected by a number of determinants: time span between cover crop killing and N demand of the succeeding main crop; soil and weather conditions governing biomass decay and N leaching; intensity of residue incorporation into soil; amount of cover crop N; C:N ratio and other chemical compounds of the biomass. In our study, the C:N ratio of rye biomass was close to or below a C:N ratio of 15–25, which was identified as threshold range delineating net mineralization from net immobilization of N (Thorup-Kristensen et al., 2003; Dabney et al., 2010). Thus, N immobilization by rye seems unlikely here. Nevertheless, Thomsen et al. (2016) reported a close negative correlation between NetN-Min and the C:N ratio in the range of 10–25 for radish cover crop biomass, indicating at least a slower release of N bound in biomass with a higher C:N ratio.

At Iho18/19, after cover crops, NetN-Min in the period March–July/August was substantially higher than in the period July/August-September/October. Given the similar NetN-Min in March–July/August among cover crops, and the low and similar NetN-Min in July/August-September/October, the whole season NetN-Min was very close for all cover crop treatments at Iho18/19. In contrast, at Goe18/19, NetN-Min after the cover crops was similar for the two time periods. This contrast between the two site/year combinations regarding the contribution of the two periods to whole season NetN-Min might have been caused by large differences between sites in the temperature regime, while differences regarding the amounts of rainfall were less pronounced. Cover crops caused at least a trend toward higher N mineralization than fallow in the first period until July/August, while in the second period N mineralization was lower. Thus, apparently, net mineralization of cover crop N occurred in the first period while in the second period net immobilization was predominant.

At Goe19/20, NetN-Min for March–July/August was positively correlated with both cover crop C and N content, which was primarily caused by the high values after radish and rye where N_min_ in March was low and increased strongly thereafter. The high initial NetN-Min was likely due to overwinter survival of cover crops at this site/year combination, causing both low N_min_ in March and fast mineralization of the readily decomposable fraction of the plant material in spring (Jensen et al., 2005; Sievers and Cook, 2018). For radish and rye, N content and C:N ratio was similar, and higher than values of oat and vetch, for which the initial flush of mineralization likely occurred earlier in winter after frost killing (vetch) or senescence (oat), even if temperatures were low (Froseth et al., 2022).

At Iho18/19, there was no correlation between cover crop biomass properties and NetN-Min, which might have been due to the overall low N content of cover crops with only small differences among species. Contrastingly, at Iho19/20, NetN-Min correlated negatively with cover crop C and N content for the period July/August–September/October. This might indicate N immobilization processes caused by non-leguminous cover crops as previously reported by Li et al. (2015), especially in the first years after integration of cover crops in the rotation (Constantin et al., 2011). However, at all site/year combinations of our study except Iho18/19 the C:N ratio of radish and rye was close to 10 and in between that of oat and vetch and could, thus, not explain the differences in N mineralization observed. For comparison, Jensen et al. (2005) reported an N release of up to 40% of the initial amount of residue N for plant residues with a C:N ratio of 10 compared to only 23% for material with a C:N ratio of 20 within 217 days of decomposition. However, plant biomass properties other than C:N ratio might have affected the intensity of decomposition (Thorup-Kristensen et al., 2003; Froseth et al., 2022).

Compared to NetN-Min, the parameter N_eff_ provides direct information on the sugar beet N supply from cover crop cultivation compared to fallow. It includes the effect of differences in soil N_min_ at the beginning of the growing season but excludes bias that might occur from changes in N_min_ during the growing season. At all site/year combinations, cover crops tended to increase N_eff_ in the first observation period by up to 50 kg ha^−1^, but pronouncedly lowered N supply in the second period by 10–100 kg N ha^−1^. From March to July/August, the lowest positive cover crop N_eff_ was calculated for rye, while from July/August to September/October differences among cover crops were not consistent. Similarly, e.g., Sievers and Cook (2018) found differences in the N release from vetch and rye plant material mainly to occur in the first 2–4 weeks after decomposition had started. Overall, the lower sugar beet N supply late in the growing season after cover crops compared to fallow hints at a certain amount of the N taken up by the cover crops not being released in time to be taken up by the first following main crop.

### Cover crop effects on sugar yield

Sugar yield was mostly not significantly different among cover crop treatments except for Goe18/19, similarly to the mostly non-significantly different N content at harvest. Thus, despite a lower N availability between July/August and September/October after cover crops than after fallow and, connected to this, a lower crop growth as indicated by the lower N uptake in that period, cover crops in general mostly did not affect final sugar yield negatively. The detrimental effects in the later growth stages stemming from the apparent N immobilization by the cover crops might be counteracted by the higher mineralization at the beginning of the growing period and by other factors not related to N, such as more beneficial growing conditions at an early stage due to an improved soil structure (Haruna et al., 2020; Grunwald et al., 2022).

Regarding differences between the cover crops, cover crop biomass properties revealed meaningful relations to sugar yield at Goe18/19 and Iho19/20 with negative correlation coefficients for cover crop C and N content, which was mainly related to the lower sugar yield after radish and rye. As discussed previously, cover crop C and N contents were negatively correlated with spring N_min_ at these two site/year combinations, which in turn was positively correlated with sugar yield. This relation was much closer when excluding the fallow treatment in the correlation analysis. At Goe19/20, however, there was no correlation between spring N_min_ and sugar yield. At this site, the overall highest sugar beet N content, NetN-Min and sugar yield was measured across all trials with smaller differences among treatments than at the other site/year combinations, indicating that N supply or other parameters influenced by cover crop growth were likely not limiting beet growth. When regarding the N effect of cover crops more specifically, mineralization during the growing season had no supplemental effect in addition to spring N_min_ in our experiments. For a broad range of Central European sugar beet fields, a N supply level of 130–160 kg N ha^−1^, comprising soil N_min_ in early spring und N fertilizer dose, was shown to be adequate for achieving maximum yield, provided at least 100–140 kg N ha^−1^ are mineralized during the growing season (Märländer, 1990; Allison et al., 1996), which was the case in our trials. For calculating the potential of cover crops to reduce the amount of fertilizer N needed to reach optimum N supply, experiments with increasing fertilizer N doses have to be performed. For our trials, an analysis on this aspect concerning the other three N fertilization treatments is in preparation.

## Conclusions

Our study provides a comprehensive evaluation of determinants affecting soil N_min_ in spring after cover crops differing in biomass properties. Winter-hardy rye and oil radish, even though the latter is not completely winter-hardy under Central European conditions, revealed the greatest potential for scavenging nitrate from the soil, while saia oat and spring vetch appeared to require favorable soil and weather conditions in autumn as well as overwinter periods without frost for as long as possible for conserving high amounts of N in biomass. In case of early senescence, these crops may release N during winter already, thereby increasing the risk of leaching and decreasing the potential N supply to the subsequent main crop. Thus, for effective cover cropping in Central Europe, species with a sufficiently high frost tolerance should be chosen, either as pure stands or in mixtures.

Despite large differences in cover crop biomass, whole season N supply to subsequent sugar beet was neither different among crop species nor was it positive compared to bare fallow. In consequence, considering solely cover crop N content appears insufficient for precise calculation of the fertilizer N demand of sugar beet. However, using cover crop N content, possibly derived from spectral information, in combination with local soil and weather data, modeling approaches might have the potential for a sufficiently precise site-specific estimation of (i) cover crop N mineralized and leached over winter, (ii) soil N_min_ after winter, and (iii) cover crop effects on N mineralization during spring to calculate the optimum fertilizer N dose for subsequent main crops.

Finally, we could not identify an enhancing cover crop effect on sugar beet yield; in contrast, winter rye cover crop tended to decrease yield. Thus, our results indicate that factors other than N supply from cover crops had a higher impact on sugar beet yield formation on the deep fertile Luvisol soils of our study.

## Data Availability Statement

The original contributions presented in the study are included in the article/Supplementary material, further inquiries can be directed to the corresponding author.

## Author Contributions

H-JK and RR specified the research questions and designed the experimental setup and measurements. DG and H-JK performed the statistical evaluation. H-JK developed the concept of the manuscript, prepared figures and tables, and wrote a draft, which was repeatedly revised by DG, LE, and RR. All authors contributed to the article and approved the submitted version.

## Funding

The project was supported by funds of the Federal Ministry of Food and Agriculture (BMEL) based on a decision of the Parliament of the Federal Republic of Germany *via* the Federal Office for Agriculture and Food (BLE) under the innovation support program.

## Conflict of interest

The authors declare that the research was conducted in the absence of any commercial or financial relationships that could be construed as a potential conflict of interest.

## Publisher's note

All claims expressed in this article are solely those of the authors and do not necessarily represent those of their affiliated organizations, or those of the publisher, the editors and the reviewers. Any product that may be evaluated in this article, or claim that may be made by its manufacturer, is not guaranteed or endorsed by the publisher.
