# Supplementary figures and images for: Temporal dynamics of sugar beet (Beta vulgaris L.) N supply from cover crops differing in biomass quantity and composition
Source: Front Plant Sci. 2022 Aug 4;13:920531. doi: 10.3389/fpls.2022.920531 (PMC9387348; doi:10.3389/fpls.2022.920531)

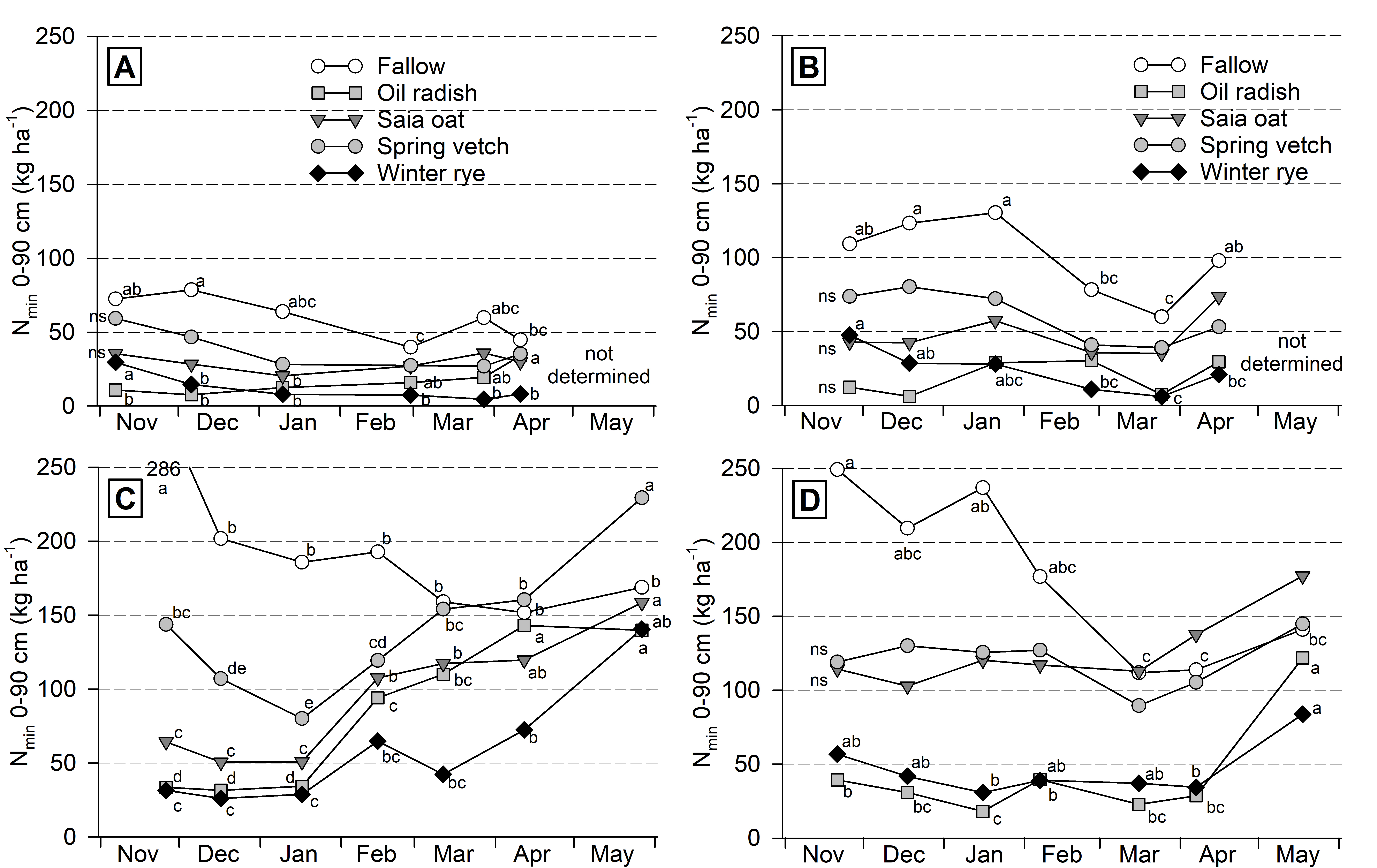

Supplement: Supplementary file 1 [file Image_1.jpg]
